# A Neural Network Model of Ventriloquism Effect and Aftereffect

**DOI:** 10.1371/journal.pone.0042503

**Published:** 2012-08-03

**Authors:** Elisa Magosso, Cristiano Cuppini, Mauro Ursino

**Affiliations:** Department of Electronics, Computer Science and Systems, University of Bologna, Bologna, Italy; The University of Plymouth, United Kingdom

## Abstract

Presenting simultaneous but spatially discrepant visual and auditory stimuli induces a perceptual translocation of the sound towards the visual input, the ventriloquism effect. General explanation is that vision tends to dominate over audition because of its higher spatial reliability. The underlying neural mechanisms remain unclear. We address this question via a biologically inspired neural network. The model contains two layers of unimodal visual and auditory neurons, with visual neurons having higher spatial resolution than auditory ones. Neurons within each layer communicate via lateral intra-layer synapses; neurons across layers are connected via inter-layer connections. The network accounts for the ventriloquism effect, ascribing it to a positive feedback between the visual and auditory neurons, triggered by residual auditory activity at the position of the visual stimulus. Main results are: i) the less localized stimulus is strongly biased toward the most localized stimulus and not vice versa; ii) amount of the ventriloquism effect changes with visual-auditory spatial disparity; iii) ventriloquism is a robust behavior of the network with respect to parameter value changes. Moreover, the model implements Hebbian rules for potentiation and depression of lateral synapses, to explain ventriloquism aftereffect (that is, the enduring sound shift after exposure to spatially disparate audio-visual stimuli). By adaptively changing the weights of lateral synapses during cross-modal stimulation, the model produces post-adaptive shifts of auditory localization that agree with in-vivo observations. The model demonstrates that two unimodal layers reciprocally interconnected may explain ventriloquism effect and aftereffect, even without the presence of any convergent multimodal area. The proposed study may provide advancement in understanding neural architecture and mechanisms at the basis of visual-auditory integration in the spatial realm.

## Introduction

The different senses are not treated as separate modules in our brain, rather they interact with one another. Hence, interpretation of data in one modality is often influenced by information from the other modalities [1]. A useful approach to investigate cross-modal interactions is to create conflict situations in which discordant information are provided by two different sensory modalities.

This approach has been largely employed to study interactions between audition and vision in spatial localization processing; in this context, conditions of audio-visual spatial conflict are imposed. When presenting an observer with an auditory stimulus and a synchronous but spatially discrepant visual stimulus, the location of the auditory stimulus is perceived shifted toward the location of the visual stimulus [2], [3]. This effect is generally known as the *ventriloquist effect* [4], [5], since its main manifestations are in speech perception, when the voice from an actual source appears to come from elsewhere. In speech perception, visual-auditory binding may be further facilitated by additional cognitive factors. However, several studies showed that visual bias of auditory location occurs not only with complex and meaningful stimuli, but also with neutral and simple stimuli, such as spots of light and tone or noise bursts [2], [6]–[8]. These studies suggest that the shift of auditory location cannot be ascribed only to cognitive factors or voluntary strategies but is due – at least partly – to a phenomenon of automatic attraction of the sound by the simultaneous and spatially separate visual input.

The ventriloquism effect is intuitively explained by the spatial dominance of vision with respect to the other senses. But how this dominance is accomplished at the neural level is still an open question.

A traditional view [5] assumes that evolution has led to an inherent advantage of visual input over non-visual inputs, regardless of the stimulus conditions; this might be implemented in the neural circuits - for example - by visual information synaptically affecting nonvisual information but not vice versa.

An alternative view proposes that the visual dominance results from a statistically optimal integration of the visual and auditory information [9]. According to this hypothesis, the brain behaves as an optimal observer that combines information derived from the noisy auditory and visual representations to infer the most likely location of the physical stimulus. In this computation, the optimal observer takes into account the uncertainty of each cue localization when deriving the combined estimate; so when one cue localization is less certain than another the estimate is biased toward the more reliable cue. According to this hypothesis, there is not an intrinsic dominance of one sense over the other, but it is the most reliable stimulus (that is, the stimulus which is best localized in space) that dominates. As visual localization is usually far superior to auditory localization, vision normally dominates. Recent experiments have strengthened this view as under appropriate conditions (i.e., by sufficiently degrading visual spatial information) the auditory stimulus may capture the spatial location of the visual stimulus [10].

Exposure to audio-visual spatial conflict – besides producing the immediate *online* displacement of sound toward the simultaneous visual stimulus (ventriloquism effect) –produces an *offline* effect, named *ventriloquism aftereffect*. That is, after a somewhat prolonged exposure to a consistent audio-visual spatial disparity, the localization responses to *unimodal* auditory stimuli are displaced in the same direction as the previously presented visual stimulus. The ventriloquism aftereffect has been demonstrated in a number of paradigms [11]–[16] and it has been ascribed to a form of rapid plasticity involving auditory cortical areas [14]–[16].

In the last decades, several studies have proposed Bayesian probabilistic models to account for the ventriloquism effect [10], [17], [18] and also aftereffect [19], [20] . Whereas these models successfully describe perceptual responses within the framework of the Bayesian inference, they lack to relate these responses to neural activity and neuron interconnections.

Connectionist models based on artificial neural networks have been proven to be powerful tools to explain perceptual and behavioural responses in terms of neural activities and architecture. In recent years, we developed neural network models investigating aspects of multisensory integration and its plasticity properties, such as audio-visual integration in the Superior Colliculus [21]–[24] and visual-tactile interaction for peripersonal space representation [25]–[27].

Here, we propose a neural network model that may contribute to elucidate the neural mechanisms at the basis of the ventriloquism effect and aftereffect. Complexity of the model is intentionally maintained at a minimum level, in order to provide a simplified - although biologically plausible - scenario where the role of the involved mechanisms can be easily outlined and understood. The model consists of two chains of unisensory neurons, visual and auditory respectively. Neurons communicate via lateral intra-layer synapses and via inter-layer synapses. The only a-priori difference between the two layers concerns the spatial resolution (i.e., the width of the receptive field), the auditory resolution being smaller than the visual one. The model demonstrates that this assumption explains the main features of ventriloquism effect and produces – when the lateral intra-layer synapses are subjected to Hebbian plasticity – post-adaptive shifts in auditory localization (ventriloquism aftereffect).

## Results

Two sets of results are presented. First, the model was used in its basal (i.e., pre-training) configuration to simulate conditions leading to ventriloquism effect. Second, the model was trained via Hebbian learning rules that modify intra-layer synapses, and then tested in the after-training configuration to assess its ability to reproduce ventriloquism aftereffect. In this section, short qualitative descriptions of the neural network and Hebbian rules anticipate presentation of results. A quantitative description with all equations and parameter assignment can be found in “Materials and Methods” section.

### The Basal Model

**Figure 1:**
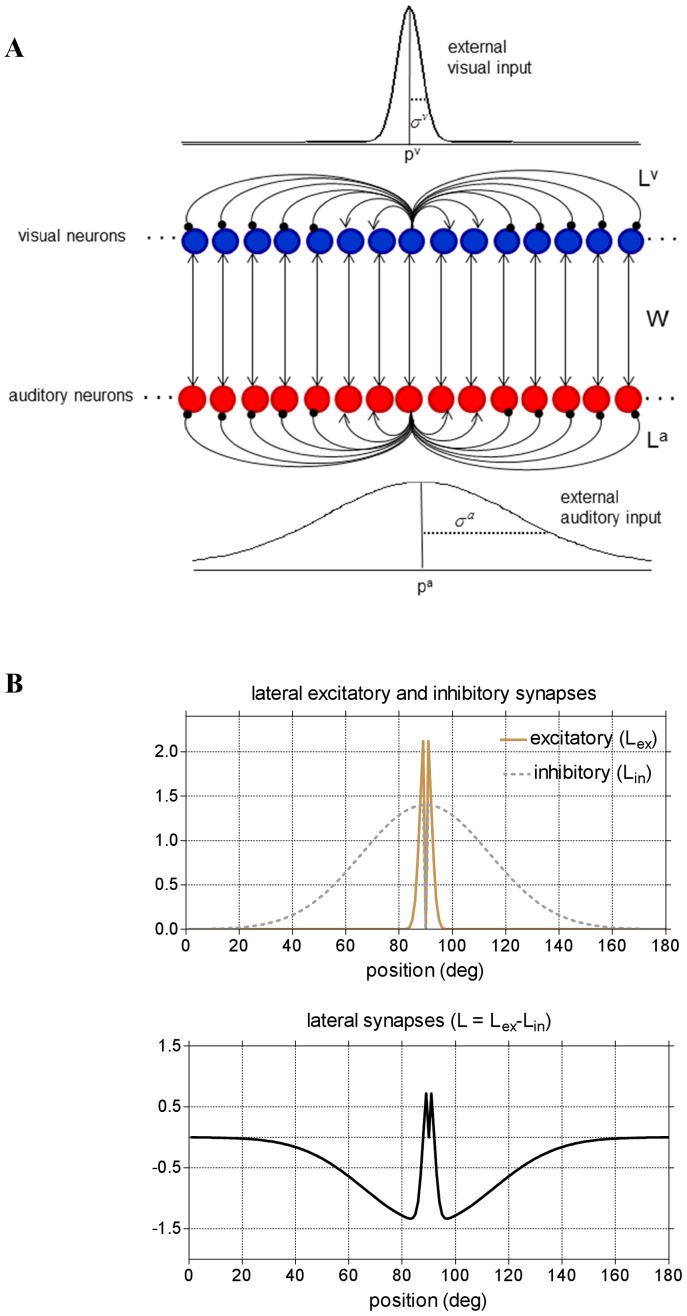
Overview of network architecture. (**A**) Schematic diagram of the neural network. Each red (blue) circle represents an auditory (visual) neuron. Each line represents a synaptic connection: lines ending with an arrow indicate excitatory connections; lines ending with a solid point indicate inhibitory connections. The Gaussian patterns mimic the external visual and auditory inputs; the Gaussian functions are centered at position *p^m^* (*m = v* visual, *m = a* auditory), which represent the location of stimulus application, and have standard deviation *σ^m^* and strength 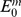. The fundamental assumption is *σ^a^*>*σ^v^*. Neurons between layers are connected via excitatory inter-area synapses (strength *W*). Neurons within each layers are connected via lateral (excitatory and inhibitory) synapses. For simplicity, only lateral synapses emerging from one neuron are displayed. In basal conditions, each neuron receives and sends symmetrical lateral synapses. (**B**) Pattern of the lateral synapses targeting (or emerging from) an exemplary neuron in either layer, in pre-training condition. Lateral excitatory (*L_ex_*) and inhibitory (*L_in_*) synapses have a Gaussian pattern with excitation stronger but narrower than inhibition. Auto-excitation and auto-inhibition are excluded. Net lateral synapses (*L*) are obtained as the difference between excitatory and inhibitory synapses and assume a “Mexican hat” disposition.

The model consists of two chains of *N* auditory and *N* visual neurons (*N* = 180), respectively (Fig. 1). Each neuron codes for information at a specific position of space, and all neurons are topologically aligned, i.e., proximal neurons in the array code for proximal positions in space. We assumed a distance of 1° between adjacent neurons, so that each layer covers an area of 180° in the visual and acoustic space. Neuron response is described with a first order differential equation, that simulates the integrative properties of the cellular membrane, and a steady-state sigmoidal relationship, that simulates the presence of a lower threshold and an upper saturation for neural activation. The saturation value is set at 1, i.e., all activities are normalized to the maximum.

Neurons within each layer are connected via *lateral* synapses; moreover neurons in the two layers are reciprocally connected via *inter-area* synapses. Hence, the net input that reaches a neuron is the sum of three contributions: an *external* input, a *lateral* input coming from other neurons in the same unisensory area, a *cross*-*modal* input from neurons in the other modality.

The visual and auditory inputs are represented as Gaussian functions that mimic spatially localized external stimuli (such as “beeps” and “flashes”), filtered by neurons receptive fields (RFs). The central point of the Gaussian function corresponds to the position of stimulus application in the external world (*p^a^* and *p^v^*, for the auditory and visual stimulus respectively); the standard deviation of the Gaussian function (*σ^a^*, *σ^v^*) is related with the width of neurons RF. A fundamental assumption of the present model is that the visual RF is much smaller than the auditory one, i.e. *σ^v^*<*σ^a^*_._

The lateral input originates from lateral connections within the same unimodal layer. These include both excitatory and inhibitory lateral synapses. Before training they are arranged with a classical Mexican-hat disposition (a central excitatory zone surrounded by an inhibitory annulus, see Fig. 1): thus, each neuron excites (and is excited by) its proximal neurons, and inhibits (and is inhibited by) more distal neurons. Hence, distal stimuli of the same modality tend to suppress reciprocally (i.e., interact via a competitive mechanism).

The cross-modal input is computed assuming that neurons of the two areas are reciprocally connected via one-to-one excitatory synapses. Hence, a neuron receives excitation only from the neuron of the other modality placed at the same spatial position.

Except for standard deviation of external input (see above), all other parameters have been set at the same basal value in the two layers (see Table 1 for parameter values and section “Materials and Methods” for parameter assignment criteria).

**Table 1:** Basal value of model parameters.

**External stimuli**	*E_0_* = 15	*σ^v^* = 4 deg	*σ^a^* = 32 deg		
**Individual neuron response**	*θ* = 12	*s* = 0.6	*τ_y_* = 3 ms		
**Synaptic connections**	*L_ex0_* = 2.4	*σ_ex_* = 2 deg	*L_in0_* = 1.4	*σ_in_* = 24 deg	*W* = 5
**Hebbian rules**	*α_ex0_* = 0.015	*α_in0_* = 0.025	*θ_post_ = *0.5	*L_max_* = 2.4	

Symbols without superscript *m* (*m* = *a*, *v*) denote parameters that assume the same value in the visual and auditory layers.

To assess network behavior in terms of stimuli localization, we need a quantity which represents the perception of the stimulus location from neuron population activity. We adopted the population vector metric [28], [29] in which each neuron provides a vector with magnitude corresponding to its firing rate and direction corresponding to twice its preferred position; then, these vectors are summed up and half the direction of the final vector signals the perceived position of the stimulus (see “Materials and Methods” for more details). Two alternative methods (the barycenter method and the winner-takes-all method) were tested (see Material and Methods and Fig. S1 in Supporting Information)

### Basal model results

All results are obtained by applying external stimuli starting from the resting (no stimulation) condition and maintaining them constant throughout the entire simulation. Network response was evaluated at the new-steady-state condition reached by the network. If not otherwise specified, the visual and auditory inputs have the same strength (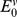 = 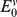 = 15) and different standard deviations (*σ^v^* = 4°, *σ^a^ = *32°) , as listed in Table 1. The position of the auditory and visual stimulus application is denoted as *p^a^* and *p^v^*, respectively.

#### Unimodal stimulation

**Figure 2:**
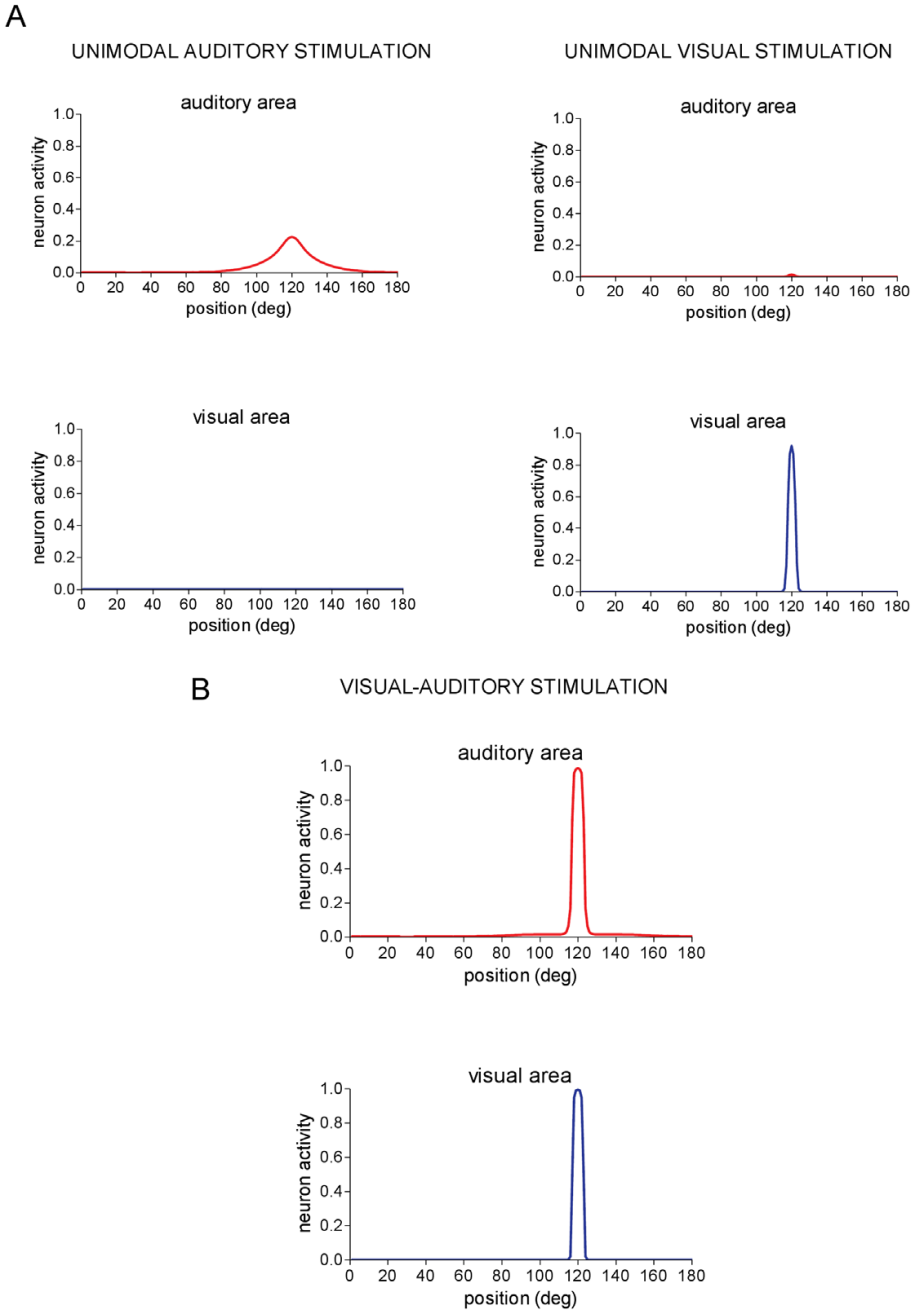
Network response to unimodal stimulation and to cross-modal spatially coincident audio-visual stimulation. (**A**) A unimodal stimulation was applied to the network and maintained constant throughout the entire simulation. Neural activity is shown in the new steady-state reached by the network. *Left panels* - Neuron activity in the auditory and visual areas in response to an auditory stimulus of amplitude 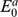 = 15 applied at position *p^a^* = 120°. No activity is elicited in the visual area. *Right panels* - Neuron activity in the auditory and visual areas in response to a visual stimulus of amplitude 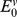 = 15 applied at position *p^v^* = 120°. No significant activity is elicited in the auditory area. (**B**) An auditory stimulus and a visual stimulus are simultaneously applied at the same spatial position (*p^a^* = *p^v^* = 120°) and maintained constant throughout the simulation. Network response is shown in steady-state condition. Auditory and visual stimuli have the same strength (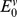 = 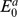 = 15). Strong reinforcement and narrowing of auditory activation occurs (compare with Fig. 2A, left panels).

First, the network was stimulated with a single unimodal (auditory or visual) stimulus to check the absence of phantom effects (i.e., to check that a single stimulus in one modality does not induce an appreciable response in the other modality). Results are displayed in Fig. 2A. Each stimulus produces a population activity centered on the position of stimulus application; population activity is broader and lower in the auditory area as a consequence of the larger input combined with lateral inhibitory competition. Worth noticing is that the model, with basal parameter values, responds to a single unimodal stimulus without any phantom in the other modality.

#### Cross-modal stimulation. Spatially coincident stimuli

Fig. 2B shows network response when a visual stimulus and an auditory stimulus were simultaneously applied at the same spatial position (*p^v^* = *p^a^* = 120°). Due to the inter-area excitatory connections, activities in the two areas reinforce reciprocally; as a main consequence, auditory area exhibits a stronger and narrower activation with respect to unimodal auditory stimulation (compare with Fig. 2A). This result agrees with data observed in vivo that auditory detection and localization is enhanced by a concomitant spatially congruent visual stimulus [30].

#### Cross-modal stimulation. Spatially disparate stimuli: the ventriloquism effect

**Figure 3:**
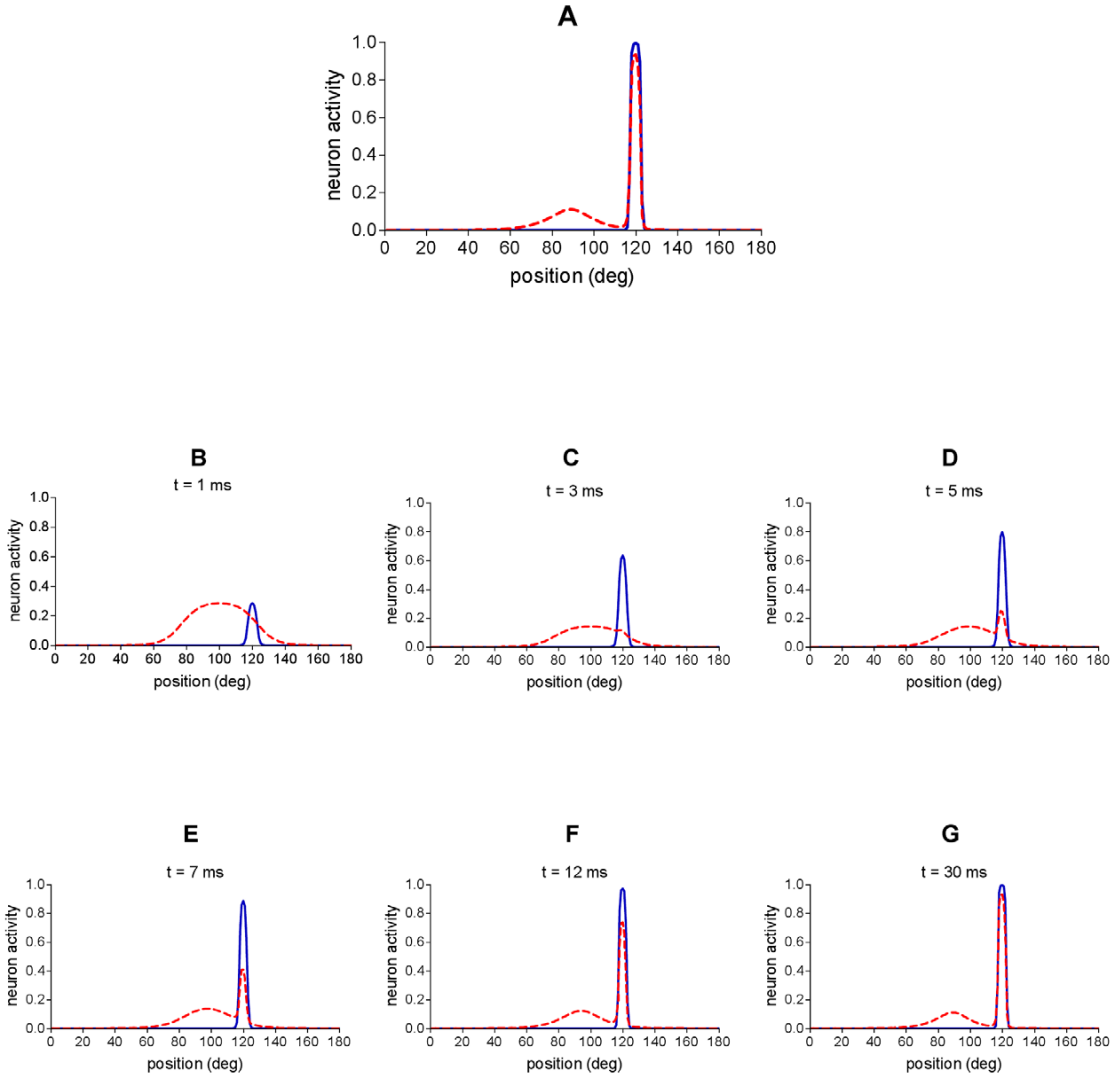
Network response to audio-visual stimulation with spatially disparate stimuli. An auditory stimulus and a visual stimulus are simultaneously applied at two different spatial positions (*p^a^* = 100°, *p^v^* = 120°) and maintained constant throughout the simulation. Auditory and visual stimuli have the same strength (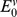 = 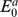 = 15). Dashed red line represents activity in the auditory area; continuous blue line represents activity in the visual area. (**A**) Network activity in the final steady-state reached by the network. (**B–G**) Different snapshots of network activity during the simulation. First snapshot (B) depicts network activity immediately after the stimuli presentation; last snapshot (G) corresponds to the final state reached by the network.

A visual stimulus and a simultaneous auditory stimulus, in disparate spatial positions (*p^v^* = 120° and *p^a^* = 100°), were presented to the network. The steady-state response in the two layers is displayed in Fig. 3A. Whereas visual activation is unaffected by the auditory stimulus, activation in the auditory area is remarkably biased towards the position of the visual stimulation. The perceived position of the auditory stimulus (computed with the vector metric) is 108.6°, resulting in a perception shift (perceived position minus original position) as great as 8.6°. In order to better understand the nature of this phenomenon, panels from B to G in Fig. 3 show different snapshots of neural activity in the auditory and visual areas at different instants in time, during the presentation of the two stimuli. Immediately after the presentation of the two stimuli (snapshots B and C), a large portion of the auditory network is activated, with a maximum of activity centered at the location *p^a^* of the auditory input; conversely, just a small number of visual neurons is active. This is the consequence of the assumed RFs of the two areas. It is worth noting that the auditory neuron located at the position *p^v^* is moderately active, whereas the visual neuron located at the position *p^a^* does not show any appreciable activation. Then (snapshot D), a positive feedback reinforcement occurs – via inter-area excitatory connections - between the visual and the auditory stimuli at the position *p^v^*; this rapidly leads to a strengthening of the auditory activity in that position which competes via lateral inhibition with the original auditory activity at position *p^a^*. When the auditory activity at position *p^v^* becomes stronger (snapshots F and G), it almost completely abolishes the auditory activity at the surrounding positions, leaving just some residual activity at greater distances.

**Figure 4:**
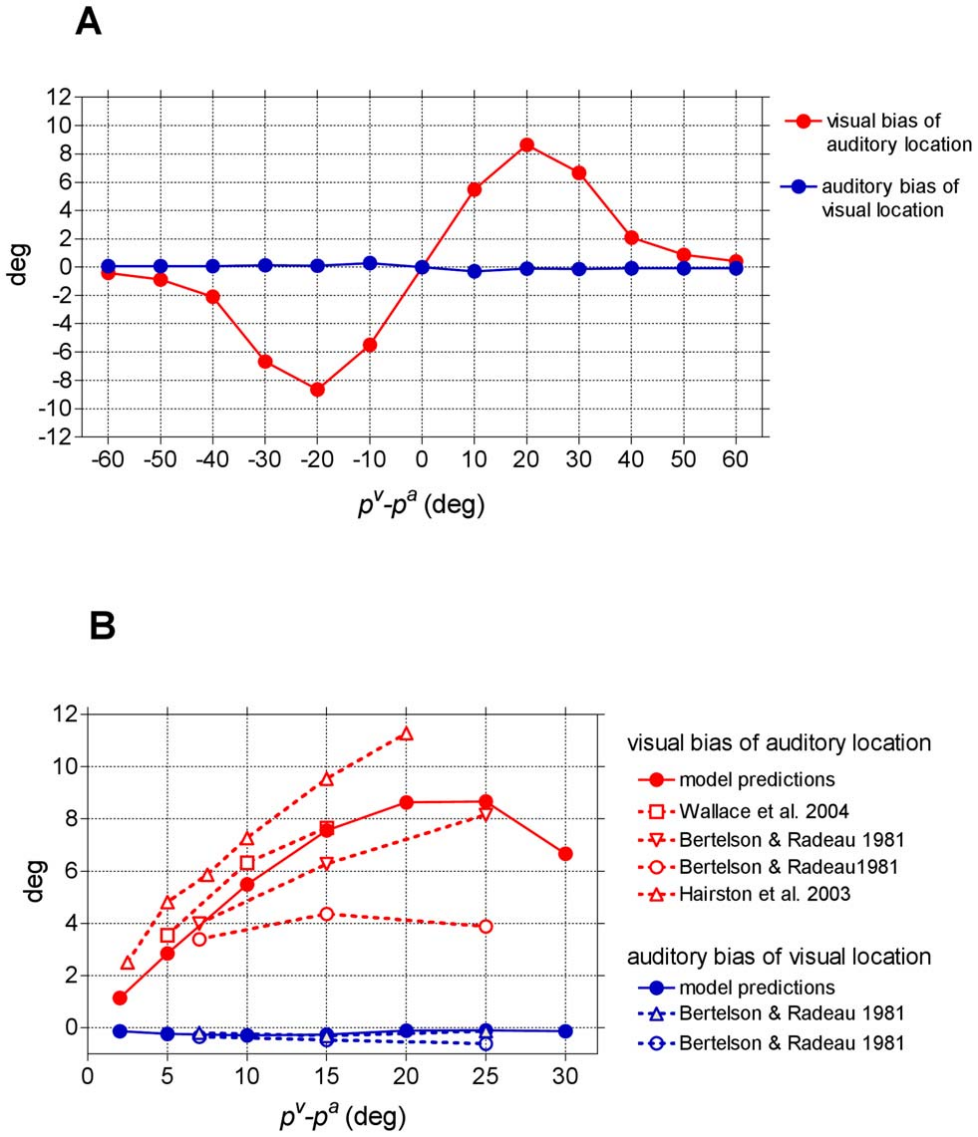
Visual bias of auditory location and auditory bias of visual location. (**A**) Biases predicted by the model - computed as perceived stimulus location minus original stimulus location – are displayed as a function of the angular separation between the location of the visual stimulus and the location of the auditory stimulus. The biases were computed with the vector metric when the network was in the new steady-state condition reached following stimuli presentation. The visual stimulus was maintained fixed at position *p^v^* = 120°, while the position of the auditory stimulus was ranged between 60° and 180° (visual-auditory angular separation ranging between −60° and +60°). In each simulation, stimuli have the same strength (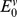 = 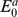 = 15). (**B**) Comparison between model predictions and in-vivo data. Biases predicted by the model (same results as (A)) are zoomed between 0° and 30° of visual-auditory angular separation for comparison with in-vivo data.

In the subsequent simulations, we investigated how the two stimuli influence reciprocally by changing their angular separation, as frequently done in in-vivo studies. In particular, the visual stimulus was maintained in a fixed position (*p^v^* = 120°), while the position of the auditory stimulus (*p^a^*) was varied from 60° to 180°. Fig. 4A shows the shift in the perception of the auditory stimulus and of the visual stimulus (i.e., the difference between the perceived position and the original position of the stimulus) computed with the population vector metric in steady–state conditions (at the end of any transient response) as a function of the distance *p^v^*–*p^a^*. If the two stimuli are far in space (above 40° distance), they behave as individual stimuli without any appreciable interaction. If the two stimuli are placed at a moderate distance (below approximately 35°), the auditory stimulus is displaced toward the visual one (ventriloquism effect), showing a shift as large as 7°–9° at visual-auditory separation between 15°–30° (similarly to what occurs in Fig. 3). Conversely, perception of the visual stimulus exhibits a very mild shift in the direction of the auditory stimulus, lower than 0.3°–0.4°. Values predicted by the model are within the range of behavioral data [2], [6], [8], as shown in Fig. 4B.

Results presented in Fig. 4 were obtained with the population vector metric. When we tested the other two metrics (the barycenter and the winner-takes-all metric), we found that the barycenter method provides results similar to the vector metric, whereas the winner-take-all metric provides unreliable values of ventriloquism (see Fig. S1 in Supporting Information). This justifies our adoption of the vector metric.

#### Sensitivity analysis

**Figure 5:**
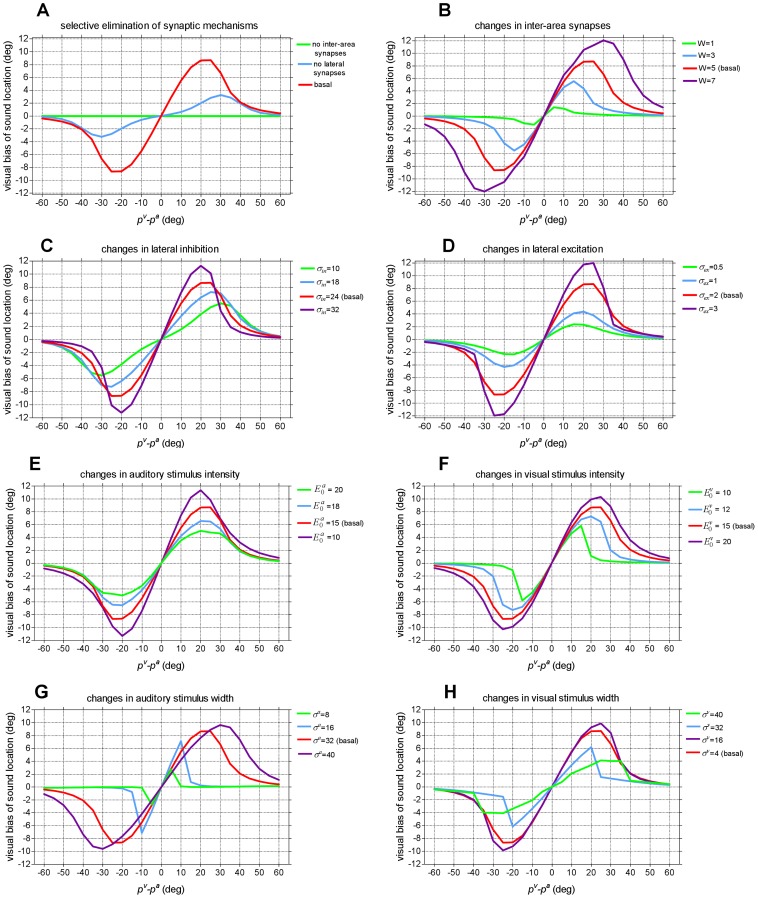
Results of sensitivity analysis. Visual bias of sound location as a function of the visual-auditory angular separation (same simulation as Fig. 4), obtained using different values for the parameters characterizing synaptic connections (panels A, B, C, D) and external stimuli (panels E, F, G, H). One parameter at a time was changed, by maintaining the others at their basal value. (**A**) Selective elimination of synaptic mechanisms (elimination of inter-area synapses, elimination of lateral synapses). (**B**) Changes in the weight of inter-area connections (*W*). (**C**) Changes in the extension of lateral inhibitory synapses (*σ_in_*). (**D**) Changes in the extension of lateral excitatory synapses (*σ_ex_*). It is worth noting that here the balance between lateral excitation and inhibition was varied by modifying the width of lateral synapses. Similar results can be obtained by acting on the strength of lateral synapses (parameters *L_ex0_*, *L_in0_*). (**E**) Changes in the strength of the auditory stimulus (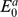). (**F**) Changes in the strength of the visual stimulus (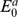). (**G**) Changes in the width of the auditory stimulus (*σ^a^*). (**H**) Changes in the width of the visual stimulus (*σ^v^*).

The previous results were attained assuming specific model parameters (see Table 1). However ventriloquism is a robust property of the model, when tested with different parameters values. Fig. 5 shows visual bias of sound location (same simulation as in Fig. 4) obtained by changing the value of one parameter at a time, while maintaining the others at their basal setting.

Results may be summarized as follows. i) (Panels A, B, C, D) – Inter-area synapses are critical for ventriloquism effect to occur: their elimination totally nulls ventriloquism and their augmentation enhances the amount of sound shift. Conversely, lateral synapses are not essential for ventriloquism to occur: mild shift of sound location is still present when they are removed. However, the amount of ventriloquism increases if the extension of the lateral synapses is augmented, reflecting a wider competition between unimodal neurons. ii) (Panels E and F) - Visual bias of sound location persists (although to a lesser extent) even when the strength of the visual stimulus is reduced or the strength of the auditory stimulus is increased. Increasing the intensity of the visual stimulus enhances the effect. ii) (Panels G and H) – The shift of sound location increases as the width of the auditory stimulus (i.e. ambiguity in auditory localization) increases. Furthermore, a less localized visual stimulus still attracts the sound until visual localization is better than auditory localization. When visual spatial information is further degraded (the visual stimulus is blurred over a large region of space) vision does not dominate over audition, rather the sound may even capture the visual stimulus. In particular, when the two stimuli have the same width (same localization ambiguity, *σ^v^* = *σ^a^* = 32°), neither sense dominates and the two stimuli attract reciprocally by the same extent (see Fig. S2A in Supporting Information). When the width of the visual stimulus overcomes the width of the auditory stimulus (e.g. *σ^v^* = 40°, *σ^a^* = 32°), a reverse ventriloquism occurs characterized by a moderate visual bias of auditory localization and a strong auditory bias of visual localization (see Fig. S2B in Supporting Information).

### Model Hebbian Rules

In order to explain the ventriloquism aftereffect, we assumed that lateral synapses are plastic and can be trained during experimental trials. In particular, we adopted a Hebbian rule with a threshold for the post-synaptic activity: excitatory synapses increase (up to a maximum saturation value) and the inhibitory synapses decrease (down to zero) in case of correlated input-output activity, provided that post-synaptic activity overcomes a given threshold. Furthermore, as often adopted in the neurocomputational literature [31], we implemented a normalization rule: the sum of excitatory and inhibitory synapses entering a given neuron must remain constant. Hence, if some excitatory synapses increase, other excitatory synapses must be depressed to maintain a constant excitatory synaptic input; similarly, if some inhibitory synapses decrease, other inhibitory synapses must be augmented to maintain a constant inhibitory synaptic input.

### Training paradigms and trained model results

Network was trained starting from its basal configuration (parameter values as in Table 1). Different simulations were performed consisting in exposing the network to repeated stimulations (in unimodal or cross-modal condition) during which lateral synapses in each layer are subjected to plasticity. Then, the aftereffects were assessed by presenting unimodal (or cross-modal) stimuli to the trained network and computing the shift in the perceived location.

#### Unimodal training

First, we checked that exposing the network to repeated unimodal stimulation (either visual or auditory) does not induce an appreciable aftereffect. Lateral synapses in the stimulated area may exhibit mild changes, but no aftereffect occurs in this condition.

#### Cross-modal training

The model was trained with repeated cross-modal stimuli, in constant spatial relationship. Two kinds of experiments were performed; for each of them we examined the effects of network exposure to spatially disparate stimuli and to spatially coincident stimuli. In the following, we will comment only the aftereffects in the auditory modality, as no visual aftereffect was presented by the trained network.

#### Training paradigm 1: Two cross-modal stimuli with assigned spatial difference and fixed locations. Case 1.a: Spatially disparate stimuli (audiovisual spatial difference = 20°)

In these trials, an auditory stimulus at position *p^a^* = 100° and a visual stimulus at *p^v^* = 120° were repeatedly given to the network. The duration of each trial was 200 ms and a total of 10 trials was performed; during each trial, stimuli were maintained constant and lateral synapses were trained.

**Figure 6:**
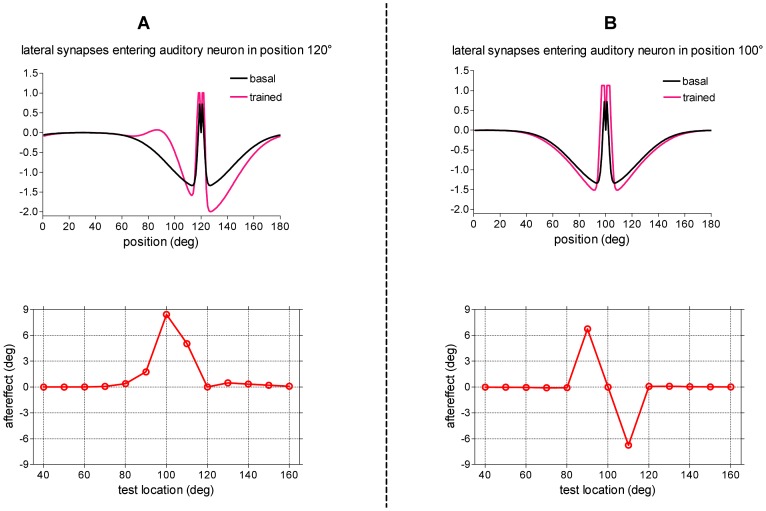
Results of training paradigm 1. (**A**) Case 1.a: training with spatially disparate stimuli in fixed position (*p^v^* = 120°, *p^a^* = 100°). *Upper panel*: Lateral synapses entering the auditory neuron in position 120° before and after training. *Lower panel*: Behavior of the trained network in response to auditory unimodal stimulation. The test auditory stimulus had strength 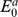 = 15, and was applied at different positions. For each position of the test stimulus, the shift in sound localization (perceived stimulus location minus original stimulus location) was computed in steady-state condition (after the transient response was exhausted) and reported as a function of the actual location of the test auditory stimulus (aftereffect). (**B**) Case 1.b: training with spatially coincident stimuli in fixed position (*p^v^* = 100°, *p^a^* = 100°). *Upper panel*: Lateral synapses entering the auditory neuron in position 100° before and after training. *Lower panel*: Behavior of the trained network in response to auditory unimodal stimulation. The same unimodal auditory test as panel A was performed to compute the aftereffect.

The main consequence of synapses training is that the auditory neurons at position close to *p^v^* = 120° (which are strongly active due to the ventriloquism effect, see Fig. 3) receive strengthened lateral synapses especially from neurons located around *p^a^* = 90° (which maintain residual activity due to the original auditory stimulus, see Fig. 3). At the same time, the normalization rule causes a decrease (i.e., less excitation, greater inhibition) of the synapses coming from inactive neurons located at positions greater than *p^v^* = 120°. The pattern of lateral synapses entering the auditory neuron at position *p^v^* = 120°, at the end of training, is presented in Fig. 6A-upper panel: this neuron is now excited (instead of being inhibited) by distal neurons located at about 30° on the left, and is more strongly inhibited by neurons located at its right.

The behavior of the trained network was tested using a unimodal auditory stimulus placed at different positions; the location of the testing stimulus was varied between 40° and 160° in 10° steps and the shift in sound location (perceived location of the auditory stimulus minus original location) was computed as a function of the test location (Fig. 6A-lower panel). Auditory stimuli located at the left of position 120° (around 80°–110°) exhibit a significant rightward shift, since now they generate more excitation at their right. Auditory stimuli located at the right of position 120° (around 125°–140°) exhibit a small rightward shift since now they generate more inhibition at their left. Worth noting is that the auditory shift remains localized in the region of space where the auditory and visual stimuli were originally presented during the training period, and does not extend to the entire space, in agreement with observations by Bertelson et al. [11].

#### Case 1.b: Spatially coincident stimuli (audiovisual spatial difference = 0°)

In these trials, the network was repeatedly exposed to a visual stimulus and an auditory stimulus at the same fixed spatial position *p^a^* = *p^v^* = 100°. Results are presented in Fig. 6 panels B.

After training, the auditory neuron at position 100° receives reinforced excitatory synapses by nearby neurons (that were strongly activated during training, see Fig. 2B), and slightly strengthened inhibitory synapses by more distant neurons (silent during training, see Fig. 2B), as depicted in Fig. 6B – upper panel. The trained network was tested by presenting a single auditory stimulus at different positions between 40° and 160° in 10° steps. At variance with case 1.a, an auditory stimulus located nearby the trained position, both at its left or right (within ±10°), is now significantly attracted towards that position (Fig. 6B-lower panel). At more distant locations no shift occurs.

#### Training paradigm 2 - Two cross-modal stimuli with assigned spatial difference but variable locations. Case 2.a: Spatially disparate stimuli (audiovisual spatial difference = 20°)

In these trials, the networks was trained with an auditory stimulus in variable position (*p^a^*, spanning from 20° to 180° in 20° increments, i.e. nine possible positions) joined with a simultaneous visual stimulus presented in a consistent spatial relationship (i.e., constant spatial difference: *p^v^* = *p^a^*+20°). The overall training procedure consists of ten trials. In each trial, the nine different positions were trained once, in a random order. Training of each position lasted 200 ms; at the end of the overall training procedure, each position was trained ten times.

**Figure 7:**
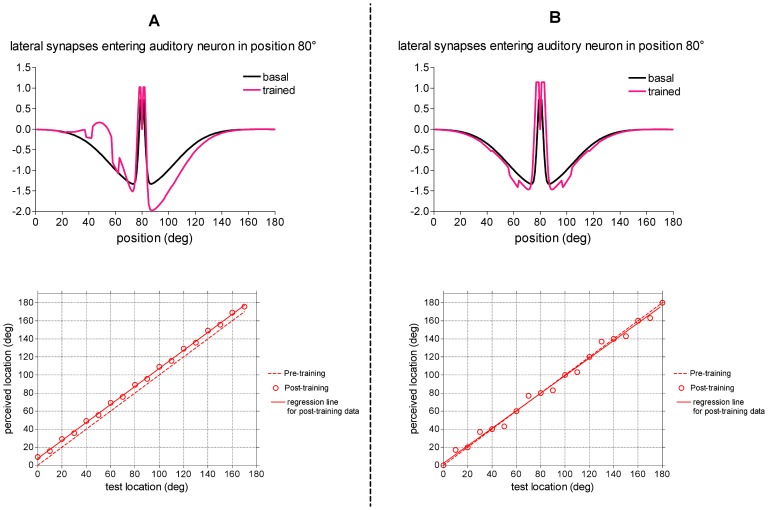
Results of training paradigm 2. (**A**) Case 2.a: training with spatially disparate stimuli in variable position with fixed audio-visual spatial disparity (20°) The auditory stimulus could be located in one among nine positions (from 20° to 180° with 20° step), and the simultaneous visual stimulus was located in fixed spatial relationship (*p^v^* = *p^a^*+20°). The overall training procedure consists of ten trials; in each trail, the nine positions were trained once (for 200 ms, each) in a random order. *Upper panel*: Lateral synapses entering an exemplary auditory neuron (neuron in position 80°, one of the trained position) are shown before and at the end of the overall training procedure. *Lower panel*: Behavior of the trained network in response to auditory unimodal stimulation. The test auditory stimulus had strength 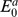 = 15, and was applied at different positions. The perceived sound location, computed in steady-state condition, was reported as a function of the original location of the test stimulus (values represented by circles). For comparison, the behavior of the untrained network was shown too (dashed line). The regression line for the post-training data (continuous line) has slope 1 and offset ∼7.5° (r^2^ = 0.9990, p<0.0001). (**B**) Case 2.b: training with spatially coincident stimuli in variable position. The auditory stimulus could be located in one among nine positions (from 20° to 180° with 20° step), and the simultaneous visual stimulus was located in the same spatial position (*p^v^* = *p^a^*). The overall training procedure was the same as panel A (but with spatially coincident stimuli). *Upper panel*: Lateral synapses entering an exemplary auditory neuron (neuron in position 80°, one of the trained position) are shown before and after the training. *Lower panel*: Behavior of the trained network in response to auditory unimodal stimulation. The same auditory unimodal test as in panel A was performed. In this case, the regression line for the post-training data is almost indistinguishable from the pre-training line.

After this training, all auditory neurons receive excitatory lateral synapses from distal neurons located approximately 20°–30° to the left, and greater inhibition from neurons located to the right; hence, all auditory neurons have asymmetrical lateral input (stronger from left, weaker from right). Pattern of the trained synapses entering an exemplary auditory neuron is shown in Fig. 7A-upper panel. This synaptic pattern results in a constant rightward shift of the perceived position of the auditory stimulus independently of the original stimulus position.

This behavior of the trained network was tested using a unimodal auditory stimulus placed at different positions from 0° to 180° in 10° increments (Fig. 7A-lower panel). Results show that an average offset as great as ∼7.5° occurs between the perceived stimulus position and the original position after training, independently of the location of the stimulus. Such network prediction is in agreement with results of behavioral studies using training paradigms similar to the simulated one [14], [15]. In particular, the magnitude of the aftereffects predicted by the model (about 37% of the cross-modal spatial disparity used during adaptation) falls within the range reported in the literature (generally, between 20% and 60% of the adapting visual-auditory disparity) [12]–[14].

#### Case 2.b: Spatially coincident stimuli (audiovisual spatial difference = 0°)

The network was trained with an auditory stimulus in variable position (*p^a^*, spanning from 20° to 180° in 20° increments) joined with a simultaneous visual stimulus in the same spatial position (*p^v^* = *p^a^*). The overall training procedure was the same as case 2.a, but with the two stimuli in spatial coincidence.

Fig. 7B-upper panel shows lateral synapses entering an exemplary auditory neuron in a trained position. Synapses modify similarly to case 1.b, that is an increase in nearby excitation associated with an increase in distant inhibition (such modifications occur in any trained position). However, in post-training conditions, sounds do not exhibit a systematic shift in auditory localization, as illustrated in the Fig. 7B-lower panel (the same auditory test as case 2.a was performed). Indeed, in this case, when the sound was located in one of the trained position, it was not displaced from that position; conversely, when the sound was located between two trained positions, it could be subjected to either a leftward or a rightward shift in a random fashion, depending on a prevalence of attraction by one of the two adjacent positions consequent to the random training. The final result is that the regression line for the post-training data is almost indistinguishable from the pre-training line. This network result agrees with data by Recanzone [15] who performed the same training paradigm as the simulated one: no systematic shift of auditory localization was observed in vivo after training, when no visual-auditory spatial disparity was present during training.

#### Audio-visual stimulation after training

**Figure 8:**
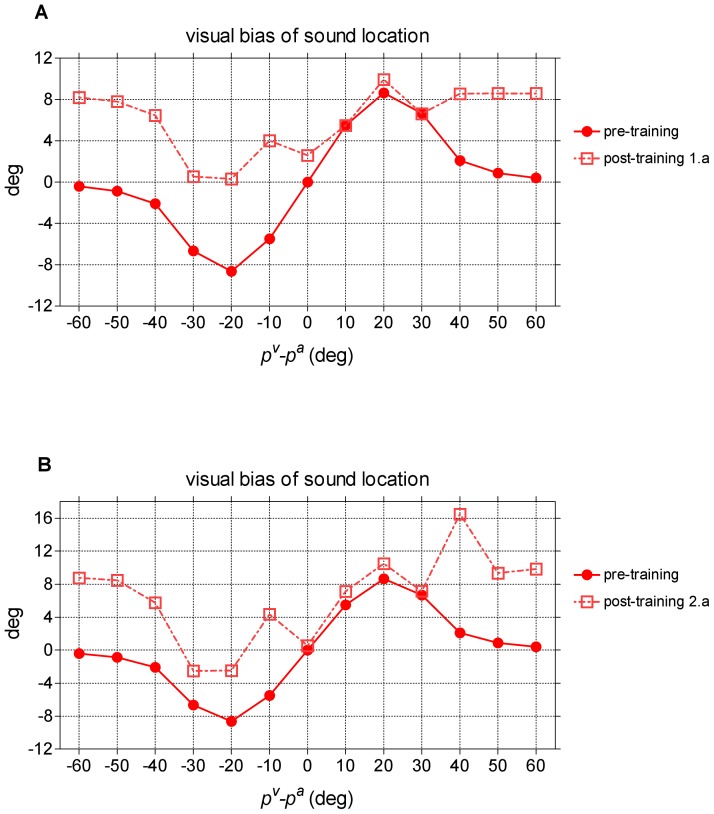
Visual bias of sound location after training. (**A**) Visual bias of sound location predicted by the model after training paradigm 1.a. The auditory stimulus was maintained fixed at position 100° (the position used during training), while the visual stimulus was located at different positions from 40° to 160° (visual-auditory angular separation ranging from −60° to 60°). The shift in sound location, computed in steady-state conditions, is displayed as a function of the visual-auditory angular separation. For the sake of comparison, results obtained before training are displayed too. (**B**) Visual bias of sound location predicted by the model after training paradigm 2.a. The same audio-visual stimulation as in panel A was performed, to compute the sound shift for different audio-visual disparities. The meaning of the symbols was the same as in panel A. Since training paradigm 2 involved all the acoustic space, the results displayed in the figure remain substantially unaltered for any position of the auditory stimulus.

A last set of simulations was performed, to test the visual bias of sound location after training. Only the trainings performed with the spatially disparate stimuli (paradigms 1.a and 2.a) were considered. An auditory stimulus at a fixed position, joined with a second visual stimulus at different positions, were given to the trained network, and the same curve as in Fig. 4 was computed (visual bias of sound location). A comparison between the two curves, obtained before training and after training with paradigm 1.a is shown in Fig. 8A. The auditory stimulus was applied at position 100° (the same used during the training), while the location of the simultaneous visual stimulus was varied from 40° to 160°. When the visual stimulus is located at a distance greater than 40° from the auditory stimulus (i.e., where a negligible ventriloquism effect occurred before training) no clear interaction occurs between the visual and the auditory stimulus, and so the sound location shift remains the same as in Fig. 6A lower panel (i.e. about 8.5° for test location in 100°): this can be entirely ascribed to the change in lateral synapses within the auditory region. When the visual stimulus is located at 120° (which was the same position used during training in paradigm 1.a) a moderate increase is evident in the sound location shift (about 10°). Conversely, if the visual stimulus is located at the left of the sound, when a leftward shift occurred before training, the sound location shift is reduced. In this condition, in fact, two opposite mechanisms are operating: i) the classic aftereffect (due to a change in lateral synapses within the auditory region) which moves the sound perception to the right; ii) the attraction by the visual stimulus, which moves sound location to the left. When the visual stimulus is at 70°–80° (20°–30° to the left of the auditory stimulus) the two effects almost completely balance.

A similar result can be obtained when the network is trained with the paradigm 2.a (see Fig. 8B); in this case, however, the alteration in sound shift is independent of the position of the auditory stimulus.

## Discussion

In this work we propose that a simple neural network, consisting of two spatially organized unimodal layers with different receptive fields and connections in spatial register can explain the ventriloquism effect. Moreover, Hebbian training of the lateral synapses within each layer can account for the ventriloquism aftereffect. The present model makes use of a minimum number of mechanisms to explain the origin of ventriloquism and the subsequent aftereffect phenomenon.

### Neural network architecture and mechanisms

In the following, neural network mechanisms and architecture will be commented and relationships with biological mechanisms outlined.

#### 1) Unimodal layers

The model assumes the presence of an auditory area and a visual area spatially organized (i.e., proximal neurons code for spatially proximal inputs), with the spatial resolution of auditory neurons smaller than the spatial resolution of the visual ones (this is the only difference between the two layers assumed a-priori in the model). Although we do not aspire to establish a definite correspondence between model areas and brain areas, some regions may be proposed as possible candidates, as also discussed in recent review papers [32], [33]. It is well known that the primary visual system contains a topographic representation of the visual space and it is characterized by a high spatial resolution, individual neurons having sharply tuned receptive fields. A role of the primary visual cortex in mediating ventriloquism is supported also by psychophysical data on human subjects (see point 2 below). Spatially sensitive neurons [34], [35] have also been found in the auditory primary area (AI); these neurons are characterized by broad spatial tuning (above 40°–60°) that can easily accommodate the ventriloquism effect. Also secondary auditory cortical areas (such as the caudomedial (CM) field) may be involved in the ventriloquism; in particular, CM field neurons have better spatial sensitivity than AI neurons [35], [36], but still lower than primary visual neurons.

An important point is that the model does not impose strict constrains on RF dimensions for ventriloquism effect to occur. Indeed, results of sensitivity analysis evidence that the ventriloquism effect is quite robust when tested with different dimensions of the RFs: even a moderate difference is sufficient for the more localized stimulus to attract the less localized one. Moreover, experimental manipulations [10] show that the auditory stimulus can capture the visual one if the visual stimulus is spatially degraded. The model can simulate this effect very well, using a spatial input for the visual stimulus wider than for the auditory one (see Fig. 5H and Fig. S2 in Supporting Information).

Finally, it is worth noting that the spatial topography adopted for each layer should be intended mainly as a functional topography rather than as a merely anatomical topography. In other words, we meant that neurons coding for proximal spatial positions - although not necessarily proximal in the anatomical space – tend to excite reciprocally and to show correlated activities (i.e. they are functionally proximal); conversely, neurons coding for different spatial positions - although not necessarily distant in the anatomical space - tend to be negatively correlated and to inhibit reciprocally (i.e., they are functionally distant). In our model, functionally proximal (distant) neurons were set at adjacent (remote) locations in each layer, as this choice largely simplifies numerical implementation; however, the model may be redesigned by removing this constraint (e.g., by disposing neurons in a random fashion within the layer, yet preserving their functional connectivity pattern) and results will still hold. In particular, this might be done for the auditory layer, as an anatomical topographic organization of spatially tuned neurons seems to be absent in the auditory cortices [35].

#### 2) Inter-area connections

A fundamental mechanism in the model is the existence of a direct excitatory link between the auditory and visual neurons coding for the same spatial position. The value of these connections was set so that a single unimodal stimulus provided in one area does not evoke any phantom activity in the other unstimulated area. The role of this mechanism becomes evident in case of cross-modal stimulation (Figs. 2B and 3): a moderate activity in the auditory area, even below the activation threshold (e.g. as in Fig. 3), is initially reinforced by the visual stimulus via the inter-area connections. Then, the simultaneous visual and acoustic activities at the same position are reciprocally amplified by the presence of a positive loop, leading to a strong reinforcement.

Multisensory interactions in unimodal (even primary) auditory and visual areas is supported by electrophysiological and neuroimaging studies [37]–[39]. For example, Martuzzi et al. [39], analyzing the BOLD signal, found a facilitation of the hemodynamic response in case of audio-visual stimulation - with respect to unisensory stimulation - both in the primary visual area and primary auditory area. Visual modulation of auditory cortex was observed during studies of audiovisual communications both in humans and monkeys [38]. Especially relevant, Bonath et al. [37] in a combined ERP-fMRI study, demonstrated that spatially discrepant auditory and visual stimuli (e.g. a central sound and a left flash) – that produced ventriloquism illusion - were associated with an activation in the auditory cortex biased toward the hemisphere contralateral to the perceived sound shift (i.e., the right hemisphere in our example).

An important question, not completely clarified in the literature yet [32], [37] is whether the audio-visual interaction occurs via a direct link between the two areas (as hypothesized in our model) or rather arises via a positive feedback from a higher multimodal area. Here, the ventriloquism effect was reproduced by implementing the first assumption. This choice, besides limiting model complexity (avoiding the introduction of a third multimodal layer), is supported by some data in the literature. First, recent works [40], [41] show that damages to the primary visual area abolished ventriloquism, whereas damages to parietal cortex did not produce any modulation of ventriloquism. These results suggest that multisensory areas in parietal cortex do not play a prominent role in ventriloquism, and emphasize the role of the primary visual area in this phenomenon. Moreover, the absence of ventriloquism effect in case of primary visual area lesion seems to exclude a prominent role of subcortical multisensory areas (such as superior colliculus) which were spared in the examined brain damage patients. Second, several studies indicate the existence of direct connections among modality-specific areas (such as visual and auditory) at an early processing stage [38], [39], [42].

#### 3) Lateral intra-area synapses

A third mechanism in the model is implemented via lateral intra-area synapses, which realize a sort of competitive mechanism: spatially proximal stimuli of the same modality reinforce reciprocally whereas more distal stimuli are inhibited. The presence of this synapse arrangement is well documented in many perceptive and motor areas [43]–[45]. According to the sensitivity analysis (Fig. 5A), lateral synapses are not obligatory to generate the ventriloquism effect: when eliminated, some ventriloquism persists due to inter-area connections. However, lateral synapses have two important roles: i) They contribute to reinforce auditory activity at visual stimulus position and to attenuate the auditory response at the original sound position, thus increasing the amount of the perceived shift. ii) The plasticity of lateral synapses constitutes the basis for the ventriloquism aftereffect.

#### 4) Synapse learning

In building the model, we assumed that only lateral intra-area synapses are trained during the ventriloquism effect through a Hebbian rule. An alternative possibility might be training the inter-area synapses still using an Hebbian paradigm. We discarded this choice since potentiation of these synapses, to explain aftereffect, would necessarily imply a phantom effect: a single auditory stimulus should evoke, via cross-modal synapses, a phantom visual stimulus at a different position; the latter, in turn, should attract the auditory activity. We are not aware of any data suggesting the presence of visual phantom activity evoked by the auditory stimulus during the test phase of ventriloquism aftereffect.

The Hebb rule we adopted in the model has two particularities that are often adopted in the neurocomputational literature and deemed to be neurobiologically plausible [31], [46], [47]. First, the Hebbian rule includes a threshold for the post-synaptic activity, ensuring that, during ventriloquism, auditory neurons at position *p^a^* (that are only slightly activated) reinforce excitation towards neurons at position *p^v^* (that area strongly activated) but not vice-versa. Second, the learning rule includes a normalization factor that warrants that inactive synapses (i.e., from silent neurons) become more inhibitory. Both these features are fundamental to reproduce data of ventriloquism aftereffect (Fig. 6 and 7).

### Comparison with previous models

The proposed approach differs from previous models by many aspects. Some previous studies interpreted results of audio-visual integration in the spatial realm within the Bayesian framework of optimal multisensory integration [10], [17], [18]. Such models are mainly conceptual; they can predict psychophysical data of audio-visual integration with good agreement, but neglect neural implementation of optimal integration. Moreover, in those studies, only the on-line ventriloquism effect was considered. Some recent papers [19], [20] used Bayesian models also to describe ventriloquism aftereffect; still, the underlying neural activity, circuitry and mechanisms are not elucidated. Some theoretical studies [48]–[50] overcame previous limitations proposing a neural implementation of optimal Bayesian integration (gain-encoding scheme [49]). In those models, main assumptions are that neural activity is corrupted by Poisson noise (so that an input produces a noisy hill of activity across the neural population) and that cue reliability is encoded in the height of the hill of activity (i.e. in the gain of the sensory input). Noisy hills with high gain entail high signal-to-noise ratios, corresponding to more reliable cues. Neural networks based on this approach were proven to perform cue integration efficiently, however they have never been applied to ventriloquism. An important point is that our model may produce ventriloquism even by encoding cue reliability only in the gain of the sensory input and not in its width (see Fig. S3 in Supporting Information). This evidences the versatility and robustness of the proposed scheme, model results holding for different ways of encoding cue reliability. However, the gain-encoding scheme would require the a priori hypothesis that the visual sensory input has a higher signal-to-noise ratio than the auditory input, without including any clear relationship with the spatial resolution of these neurons. We opted for a more straightforward approach, encoding cue reliability in the width of the input, which is directly related to the size of the receptive field. Furthermore, although our implementation does not require neural noise for reproduction of ventriloquism, we predict that inclusion of random noise would further enhance the phenomenon.

In line with our study, a recent relevant paper by Weisswange et al. [51] explores the crucial issue of learning from cross-modal stimulation experience. It addresses the question of how cue integration abilities, that seem not to be innate, can be acquired through development, so that the subject learns to weight uncertain cues according to their respective reliabilities, in agreement with Bayesian inference. Weisswange and colleagues proposed a reinforcement learning algorithm, implemented via a feedforward neural network, whose synaptic weights are updated via a gradient descendent algorithm. Similarly to our model, that model was able to replicate results of audio-visual interactions, where vision dominates when more reliable, and visual dominance is replaced by audition dominance as visual information are degraded. Despite some common points, our approach presents some original aspects with respect to that paper. First, our model tackles how multisensory integration experience may produce a long lasting and off-line effect on unisensory map representation (ventriloquism aftereffect); an issue not faced in that paper. Furthermore, our modeling approach is substantially different from that by Weisswange et al., as we proposed a recurrent neural network with feedback and feedforward connections, and Hebbian learning rules, for potentiation and depression. This implementation seems to be more close to biology than feedforward network and descendent gradient algorithm; indeed, recurrent synapses are ubiquitous in the cortex, and Hebbian forms of learning are known to be present in the brain [52], whereas neural implementation of gradient descendent learning is controversial [53]. Of course, on the other hand, paper by Weisswange et al. tackles important aspects that are neglected by ours such as developmental acquisition of integration capabilities and causal inference.

Our approach shares some features with a recent computational model proposed by Witten et al [54], that explores how synaptic plasticity can maintain spatial registry across input channels (e.g. visual and auditory), in response to a sensory misalignment. Similarly to our work, Witten et al found that, although the two input channels obey to the same hebbian learning rule, the amount of plasticity was highly asymmetric across the two input channels depending on their RFs: the channel with the weaker or broader RF exhibited most or all plasticity (e.g., auditory RF shifts whereas visual RF remains unchanged). However in that model, at variance with ours, plasticity occurs at the locus of cross-modal integration (by training synapses projecting from the input unimodal layers to an output cross-modal layer) and not within the single unimodal layer. Studies of ventriloquism effect and aftereffect in human subjects seems to exclude the participation of cross-modal areas (both cortical and subcortical) [15], [40], [41] in this perceptual change and adaptation. Our model shows that a third cross-modal area is not necessary for the ventriloquism effects and aftereffects to occur, and that only two communicating unimodal layers – characterized by different RFs and combined with synaptic mechanisms and plasticity– can explain the phenomena.

In conclusions, the present model may open new perspectives in the field of integration of cues with different degree of spatial reliability; in particular, due to the ubiquity of the proposed mechanisms in the real neural circuits, the proposed scheme may generalize to other domains and explain multisensory illusory phenomena outside of audiovisual space perception (such as audiotactile ventriloquism [55]).

### Lines for future experimental and theoretical studies

Some new investigations, both theoretical and experimental, may be suggested following the present research.

First, an interesting result of the model concerns the temporal evolution of auditory activity in response to a spatially disparate audio-visual stimulation (see Fig. 3, panels B–G). The model predicts that auditory activation initially rises at the position where the auditory stimulus is applied; then, with some delay, the visual stimulus attracts the evoked auditory activity which increases at the position of the visual stimulus, concomitantly decreasing (due to lateral inhibition) at the position of the actual auditory stimulus. This temporal pattern of auditory activation could be potentially tested in auditory cortex of animals via single cell recordings. It is worth noting that in the present work – as we mentioned below – we did not model the temporal characteristic of the visual and auditory receptive field. Hence the time instants we reported in the figure (above each snapshot) do not want to represent real time but just simulation time. Although the timing provided by the model is fictitious, we deem that the pattern of evolution of auditory activity might be a reliable prediction.

Moreover, the model provides two results (Fig. 6B and Fig. 8), for which clear data in the experimental literature are still lacking. The first concerns adaptation to spatially coincident visual and auditory stimuli in a fixed position (Fig. 6B): after training, the model predicts an attraction of the auditory stimuli towards the adaptation location. The second concerns the effect of a visual stimulus on a simultaneous auditory stimulus after adaptation to cross-modal disparity (Fig. 8): the model predicts a pattern of auditory shift that differs significantly from the pre-training condition, according to the modifications of lateral auditory synapses. Future in-vivo experiments may test for these effects: experimental results in line with these model predictions would further strengthen model architecture and mechanisms.

Neurophysiological data on auditory cortex [35] indicate that neurons both in the primary auditory cortex and in the caudomedial field (that are possible cortical sites of ventriloquism effect) exhibit better spatial sensitivity for stimulus azimuth than for stimulus elevation, in agreement with behavioral performances showing that auditory localization in azimuth is better than in elevation. This might correspond to anisotropic auditory RFs, larger in elevation than in azimuth. Based on these data, the model predicts that a visual stimulus should bias auditory localization at greater distances along elevation than along azimuth (see results of sensitivity analysis when increasing the width of the auditory RF, Fig. 5G). This aspect may be better explored by experimental data comparing ventriloquism effect in azimuth and in elevation, and by models involving two- dimensional layers of neurons (rather than unidimensional), to simulate different spatial sensitivity along the two dimensions.

Another important aspect concerns the frequency tuning function of auditory neurons. Primary auditory neurons exhibit sharper tuning function compared with other auditory areas (such as caudomedial field) having broader frequency tuning functions [56]. More complete models including frequency tuning functions for the auditory neurons may be used to investigate whether ventriloquism aftereffect can transfer across all neurons [12], [13], or remain confined within neurons with the same tuning functions [15], providing further implications on the functional site where auditory recalibration might take place.

Finally, in this work, we focused only on the spatial properties of the stimuli, without considering differences in timing between the auditory and visual response. It is well known that the auditory system has far better temporal acuity than vision, resulting in capture of the visual stimulus by the auditory one in the temporal realm [57]. This aspect may be investigated in future modeling studies, including an accurate description of the temporal responses of auditory and visual neurons.

## Materials and Methods

### Basal model equations

The model consists of a chain of *N* auditory and *N* visual neurons (*N* = 180). Neurons within each layer are topologically aligned. Adjacent neurons are assumed at a distance of 1°. Of course, a single neuron in our model should be considered representative of a population of neurons which code for a similar spatial position. Each neuron is referenced with superscripts indicating their array and subscripts that indicate their position within that array (i.e., indicating their spatial position/sensitivity). *u(t)* and *y(t)* are used to represent the net input and output of a given neuron at time *t*, respectively. Thus, 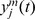 represents the output of a unit at position *j* with modality *m* (*m* = *a* or *v*, where *a* means auditory and *v* means visual).

Each neuron response is described with a first order differential equation and a steady-state sigmoidal relationship. Hence, the following differential equation can be written for a neuron with modality *m* (*m* = *a* or *v*) at position *j* (*j* = 1, 2, …, 180):


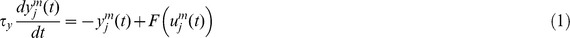


where τ*_y_* is the time constant and *F*(*u*) represents a sigmoidal relationship:


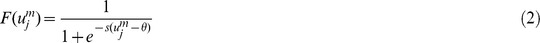


*s* and *θ* are parameters which establish the slope and the central position of the sigmoidal relationship, respectively. We used the same time constant and the same sigmoidal relationship for visual and auditory neurons, although it is possible that these neurons exhibit different characteristics (in particular, the auditory response is prompter than the visual one, i.e., auditory neurons have better temporal resolution). This choice has been adopted to reduce the number of ad hoc assumptions in the model to a minimum. Moreover, according to Eq. 2, the saturation neuron activity is set at 1, i.e., all activities are normalized to the maximum.

The net input, 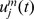 , that reaches a neuron is the sum of three contributions: an *external* input, 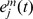, a *lateral* input coming from other neurons in the same unisensory area, 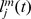,and a *cross*-*modal* input from neurons in the other modality, 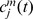.

Hence, we can write


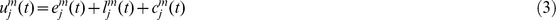


Expressions for the individual terms in Eq. 3 are given below.

#### i) The external inputs

The overall external input is mimicked using a Gaussian function, which represents the result of a local stimulus spatially filtered by the neuron receptive field. Assuming a spatially localized stimulus of modality *m* (*m* = *a* or *v*) centered at the position *p^m^* , the consequent input to the network can be written as


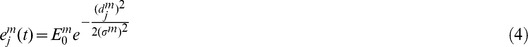


where 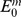 represents the strength of the stimulus, 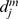 is the distance between the neuron at position *j* and the stimulus position *p^m^*, and 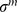 is related with the width of the RF. Elements at the extreme ends of a linear array potentially might not receive the same inputs as other units; this can produce undesired border effects. To avoid this complication, the array is imagined as having a circular structure. Hence, the following definition is used for the distance *d_j_*:


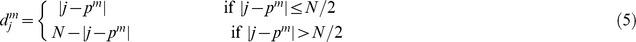


We assume that visual response has a better resolution than auditory response, that is we set *σ^v^<σ^a^*.

#### ii) The lateral input

This input originates from lateral connections within the same unimodal area. To implement this mechanism, we can write


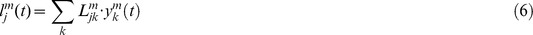


where 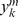 is the activity of a presynaptic neuron with modality *m* at position *k*, and 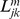 is the strength of the lateral synapse from a presynaptic neuron at position *k* to a postsynaptic neuron at position *j*, both of the same modality *m*. Lateral synapses are the difference between excitatory and inhibitory contribution, i.e.,


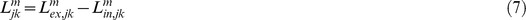


In basal conditions (i.e., before training) these synapses have a “Mexican hat” disposition, realized as the difference of two Gaussian functions. Hence


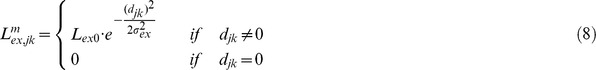



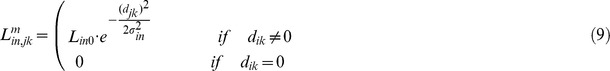


where *L_ex0_* and *L_in0_* are constant parameters which set the strength of the excitatory and inhibitory synapses; *σ_ex_* and *σ_in_* are standard deviations, which establish the rate of synapse decrease with distance, and *d_jk_* is the distance between neurons at positions *j* and *k*, defined using the same circularity as in Eq. 5, i.e.


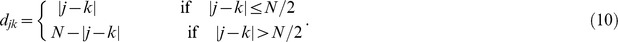


Moreover, we excluded the presence of self-loops in Eqs. 8 and 9, i.e., a neuron does not excite or inhibit itself.

Before training, lateral synapses have the same arrangement for the two unimodal areas (i.e., Eq. 7–9 do not depend on the particular modality *m*) and for all post-synaptic neurons. This choice has been adopted to fulfill a parsimony principle, i.e., to introduce a minimum number of hypotheses in the model. In order to have a Mexican Hat, one needs: *L_ex0_>L_in0_* and *σ_ex_<σ_in_*.

However, these synapses may depend on the particular modality after training (see sub-section “Hebbian training rule equations” below). In fact, we assumed that lateral synapses are plastic, hence they can be reinforced or depressed on the basis of the correlation between the pre-synaptic and the post-synaptic activities.

#### iii) The cross-modal input

For the sake of simplicity, the cross-modal input (i.e., the input that a neuron receives from neurons in the other modality) has been given a very straightforward expression. We assumed that a neuron receives excitation only from a neuron of the other modality placed at the same spatial position. Moreover, these excitations are reciprocal and have the same value (say *W*) for all neurons. Hence


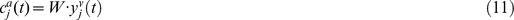



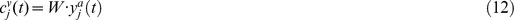


### Hebbian training rule equations

The model adopts a classic Hebbian rule for potentiation, with a threshold for the post-synaptic activity, i.e., the synapse is modified only if the post-synaptic activity overcomes a given threshold (say *θ_post_*). We have


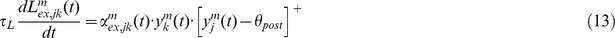



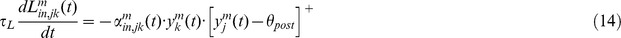


where *τ_L_* is the time constant, 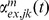 and 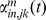 are learning factors for the excitatory and inhibitory synapses, respectively, and []^+^ denotes the function *positive part* (i.e., [*x*]^+^ = *x* if *x*≥0; [*x*]^+^ = 0 if *x*<0). According to Eq. (13) and (14) excitatory synapses increase and inhibitory synapses decrease in case of correlated input-output activity.

Two physiological constraints are imposed to the synapses: an individual saturation rule and a population normalization rule. First, each excitatory synapse cannot overcome a maximum saturation value (say *L*_max_). This is obtained assuming that the learning factor progressively decreases with the synapse strength:


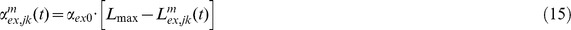


where 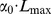 is the maximum learning factor (i.e., the learning factor when the synapse strength is zero).

Similarly, each inhibitory synapse cannot decrease below zero. Hence the learning rate decreases with the synapse value:


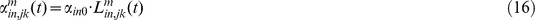


Finally, at each simulation step, all excitatory and inhibitory synapses entering a post-synaptic neuron are normalized, to maintain a constant synaptic input to each neuron. Normalization rules were applied separately for excitatory and inhibitory synapses, since these synapses make use of different neurotransmitters. We have


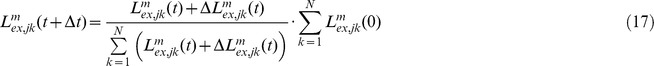



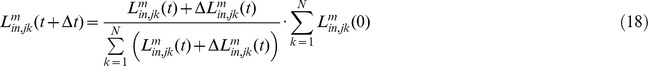


where *Δt* is the integration time step and *ΔL* denotes the synaptic change computed via Eqs. 13–16 during a single integration step.

### Computation of model outcome: perceived stimulus location

To compare model behavior with the results of psychophysical experiments, we need to compute a quantity which represents the individual perception (say *z^m^*, *m* = *a*, *v*) of the stimulus location. We tested three different metrics to calculate the individual perception of a stimulus location.

i) *The population vector metric*, according to which each neuron provides a two-dimensional vector, with its length equal to the firing rate and phase equal to twice its label. All these vectors are summed up, and the perceived orientation is taken as half the orientation of the final vector. Hence


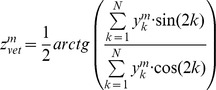


where 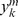 (*m* = *a*, *v*) represents the activity of the neuron at position *k* with modality *m* (*v* = visual, *a* = auditory).

ii) *The barycenter metric*, according to which the perceived stimulus location is taken as the average value (the barycenter) of the population curve. This is computed as follows:


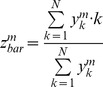


where the meaning of the symbols is the same as before.

iii*) The winner takes all metric* (WTA metric), according to which, the perceived position is provided by the neuron with the maximum response; i.e.


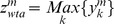


The circularity of the neurons, to avoid border effects, has been taken into account in computing the previous metrics.

Since the WTA metric provides unreliable results of ventriloquism and the barycenter metric provides similar results as the population vector metric (see Fig. S1 in Supporting Information), the latter one was adopted.

### Numerical implementation aspects

The differential equations (Eqs. 1, 13 and 17, 14 and 18) with the auxiliary equations (Eqs 2–12, 15, 16) were numerically solved within the software environment MATLAB (The MathWorks, Inc.), starting from initial null conditions and using the Euler integration method. The integration step (0.1 ms) was small enough to warrant a sufficient accuracy. Additional simulations, performed by reducing the integration step down to 0.01 ms, did not reveal appreciable differences.

To test behavior of the network before training (basal condition) and after training, we stimulated the network with one stimulus or a pair of stimuli, starting from the resting (no stimulation) condition. Stimuli were maintained constant throughout the overall simulation. Each simulation lasted sufficiently for the network to reach a new-steady state condition, at which network response, in terms of perceived stimulus position, was evaluated.

Each training trial of the network consists in the application of a stimulus or a pair of cross-modal stimuli to the network. During the overall length of each training trial (200 ms), the lateral synapses within the two layers were allowed to modify according to the learning rules.

### Parameter assignment

Basal values for model parameters (Table 1) were given on the basis of the following main criteria.

#### External inputs

As a fundamental hypothesis, the visual standard deviation (parameter *σ^v^*) was assumed smaller than the auditory standard deviation (parameter *σ^a^*) to account for the higher spatial resolution of the visual system compared with the auditory system [32], [35]. The value of *σ^v^* was assigned to produce a narrow activation in the visual area. Then, the value of *σ^a^* was assigned on the basis of data of Alais and Burr [10]. In particular, Alais and Burr - investigating dominance in visual-auditory spatial interaction - showed that when the visual blob shifted from 4° width to 32° width, behavioral results shifted from vision dominating over audition to neither modality dominating over the other (indicating that, in this case, the two modalities have the same degree of uncertain). These results suggest that a ratio *σ^a^/σ^v^* as high as 8 is consistent with significant visual biases of sound location. Furthermore, our simulation results indicate that this ratio is compatible with several data of visual-auditory interaction in the literature. However, we are aware that there may be an extreme variability in this ratio, for example depending on the specific location of the visual stimulus (central, paracentral, peripheral) [6] or on visual contrast. Regarding to this, the sensitivity analysis show that the value assigned to this ratio is not critical for ventriloquism effect to occur: indeed, even by reducing significantly this ratio (ratio = 2, *σ^v^* = 16° *σ^a^* = 32°, see Fig. 5H), the model can still reproduce visual capture of sound. Augmenting this ratio (ratio = 10, *σ^v^* = 4° , *σ^a^* = 40° see Fig. 5G) may further enhance the capture effect of vision.

The basal strength of the visual and auditory stimuli (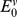 and 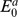) was chosen so that neuron response settles within the central part (i.e., the linear part) of the sigmoidal static characteristic. Moderate changes in these parameters might enhance or reduce ventriloquism (see Fig. 5, panels E and F), too.

#### Parameters of individual neurons

For simplicity, parameters of the static sigmoidal relationship and time constant were assumed equal for all neurons regardless of their respective area. The central abscissa (*θ*) was set to have negligible neuron activity in absence of any external stimulation. The slope of the sigmoidal relationship (parameter *s*) was assigned to have a smooth transition from silence to saturation in response to external stimuli. The value given to the time constant *τ_y_* (few milliseconds) is in agreement with those normally used in deterministic mean-field equations [43], [58].

#### Parameters of synaptic connections

Basal values of the parameters characterizing lateral synapses (*L_ex0_*, *L_in0_*, *σ_ex_*, *σ_in_*) were assigned so that: i) an external unimodal stimulus produces a region of activated neurons in the corresponding area (visual, auditory) whose extension approximately equals the dimension of the related receptive field; ii) inhibition must be sufficiently strong to warrant competition between two stimuli in the same area even at distances of 20–30 deg. Parameter characterizing inter-area synapses (*W*) was assigned to reach a compromise between the following two requirements: i) it must be sufficiently low so that an external stimulus in one modality does not induce a phantom activation in the other non-stimulated area; ii) it must be sufficiently high so that an input from one area can reinforce response of neurons in the other area when these neurons are near or just above the activation threshold.

Effects of changes in these parameters have been analyzed via the sensitivity analysis.

#### Parameters of the Hebbian rules

Learning rates for the inhibitory and excitatory synapses (parameters *α_in0_* and *α_ex0_*) were set small enough to ensure a gradual modification of synaptic pattern during the training phases. Value of the post-synaptic threshold (*θ_post_*) was assigned so that changes occur only for synapses targeting neurons activated above 50% of their maximal response. This value is sufficiently high to warrant that reciprocal synapses between two activated neurons modify asymmetrically, with the synapse targeting the more active neuron changing significantly more than the synapse targeting the less activated neuron.

Finally, the maximum strength of the excitatory synapses in basal conditions (*L_ex0_* in Eq. 8) was used as the saturation value (*L_max_*) for each excitatory synapse. That is, we hypothesized that in basal conditions, excitatory synapses that each neuron sends to the immediately near ones are already almost at their saturation value; this hypothesis is reasonable since adjacent neurons are frequently and repeatedly activated together in the daily perception of external stimuli.

## Supporting Information

Figure S1 **Comparison of alternative metrics to compute the perceived location of a stimulus starting from population activity.** We tested three different metrics to calculate the individual perception (say *z^m^*, *m* = *a*, *v*) of a stimulus location: i) *The population vector metric*, according to which each neuron provides a two-dimensional vector, with its length equal to the firing rate and phase equal to twice its label. ii) *The barycenter metric*, according to which the perceived stimulus location is taken as the average value (the barycenter) of the population curve. iii*) The winner takes all metric* (WTA metric), according to which, the perceived position is provided by the neuron with the maximum response. To compare the three metrics, the same simulations as in Fig. 4 were performed, that is the visual stimulus was maintained fixed at position *p^v^* = 120°, while position of the auditory stimulus was ranged between 60° and 180° (visual-auditory angular separation ranging between −60° and +60°). Then, the shift in the perception of the visual and auditory stimulus (difference between the perceived position and the original position) was computed, in steady-state condition, with each of the three metrics. (**A**) Visual bias of auditory location computed with the three different metrics. Results obtained with the barycenter metric and the vector metric are quite similar, with the vector metric providing just a moderately higher shift than the barycenter metric; these results are in good agreement with behavioral data (see Fig. 4B). Conversely, the WTA metric predicts much higher values of shift for moderate distances (≤30°) between the two stimuli, and no shift at larger distances (≥30°); such predictions exhibit poor agreement with behavioral data (compare with Fig. 4B). (**B**) Auditory bias of visual location computed with the three different metrics. Both the barycenter metric and the vector metric predict a mild shift of the perceived location of the visual stimulus towards the sound location, according with in-vivo data (compare with Fig. 4B); conversely, no shift is provided by the WTA metric.
(TIF)

Figure S2 **Effect of degrading visual spatial information.** This figure is at integration of the sensitivity analysis, in particular it integrates Fig. 5H. (**A**) Visual bias of sound location and auditory bias of visual location were computed when the standard deviation of the external visual stimulus equals that of the auditory stimulus (*σ^v^* = *σ^a = ^*32°). All other network parameters are maintained at their basal value. When the two stimuli are sufficiently close (distance below 20–25°), the two stimuli affect reciprocally by the same extent. (**B**) Visual bias of sound location and auditory bias of visual location were computed when the standard deviation of the external visual stimulus was set greater than that of the auditory stimulus (*σ^v^* = 40°; *σ^a = ^*32°). All other network parameters are maintained at their basal value. Sound exerts a strong capture effect on the visual stimulus, which exhibits a shift as large as 10° towards the sound location; conversely, sound is only moderately attracted by the visual stimulus.
(TIF)

Figure S3
**Alternative coding of cue reliability.** Here we tested network functioning when cue reliability is coded in the strength (rather than the width) of the input. That is, here we assumed that the visual and auditory inputs have the same standard deviation (*σ^v^* = *σ^a = ^*35°) but different strengths. All other mechanisms and parameters have been maintained unaltered. The three parts of the figure display visual (blue line) and auditory (red dashed line) activity in response to different stimulations. (**A**) Network activity in response to an unimodal visual stimulus applied at position *p^v^* = 120°. The visual stimulus had strength 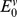 = 16 (*σ^v^* = 35°). The displayed activation refers to steady-state condition (after the transient response was exhausted). (**B**) Network activity in response to an unimodal auditory stimulus applied at position *p^a^* = 100°. The auditory stimulus had strength 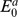 = 12 (*σ^a^* = 35°). The displayed activation refers to steady-state condition (after the transient response was exhausted). (**C**) Different snapshots of network activity at different instants during the presentation of two cross-modal stimuli in spatial disparity (*p^v^* = 120°, *p^a^* = 100°). The visual and the auditory stimuli were the same as those presented in panels A and B (that is having same widths but different strengths). The auditory activity and the visual activity tend to reinforce reciprocally owing to the inter-area synapses; due to the higher strength of the visual input, visual activity around 120° is advantaged and shows a higher increase, amplifying auditory activation at this same position. At the new steady state (t≥100 ms), the perceived position of the auditory stimulus exhibits a strong shift towards the visual stimulus location (perceived position = 109.4°), whereas the shift of the visual stimulus is moderate (perceived position = 117.5°).(TIF)
