# Supplementary figures and images for: A Neural Network Model of Ventriloquism Effect and Aftereffect
Source: PLoS One. 2012 Aug 3;7(8):e42503. doi: 10.1371/journal.pone.0042503 (PMC3411784; doi:10.1371/journal.pone.0042503)

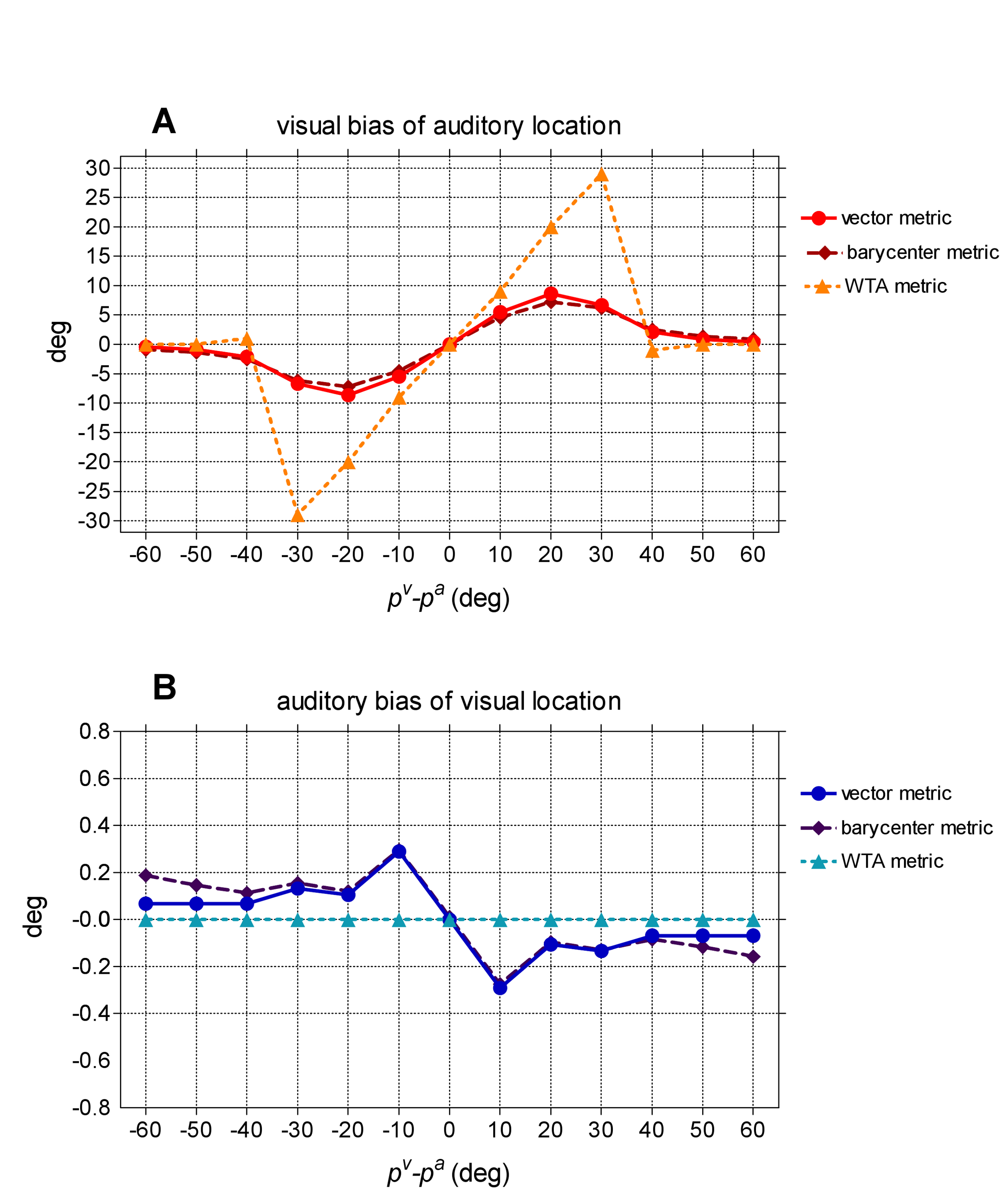

Supplement: Figure S1 — Comparison of alternative metrics to compute the perceived location of a stimulus starting from population activity. We tested three different metrics to calculate the individual perception (say zm, m = a, v) of a stimulus location: i) The population vector metric, according to which each neuron provides a two-dimensional vector, with its length equal to the firing rate and phase equal to twice its label. ii) The barycenter metric, according to which the perceived stimulus location is taken as the average value (the barycenter) of the population curve. iii) The winner takes all metric (WTA metric), according to which, the perceived position is provided by the neuron with the maximum response. To compare the three metrics, the same simulations as in Fig. 4 were performed, that is the visual stimulus was maintained fixed at position pv = 120°, while position of the auditory stimulus was ranged between 60° and 180° (visual-auditory angular separation ranging between −60° and +60°). Then, the shift in the perception of the visual and auditory stimulus (difference between the perceived position and the original position) was computed, in steady-state condition, with each of the three metrics. (A) Visual bias of auditory location computed with the three different metrics. Results obtained with the barycenter metric and the vector metric are quite similar, with the vector metric providing just a moderately higher shift than the barycenter metric; these results are in good agreement with behavioral data (see Fig. 4B). Conversely, the WTA metric predicts much higher values of shift for moderate distances (≤30°) between the two stimuli, and no shift at larger distances (≥30°); such predictions exhibit poor agreement with behavioral data (compare with Fig. 4B). (B) Auditory bias of visual location computed with the three different metrics. Both the barycenter metric and the vector metric predict a mild shift of the perceived location of the visual stimulus towards the sound lo [file pone.0042503.s001.tif]

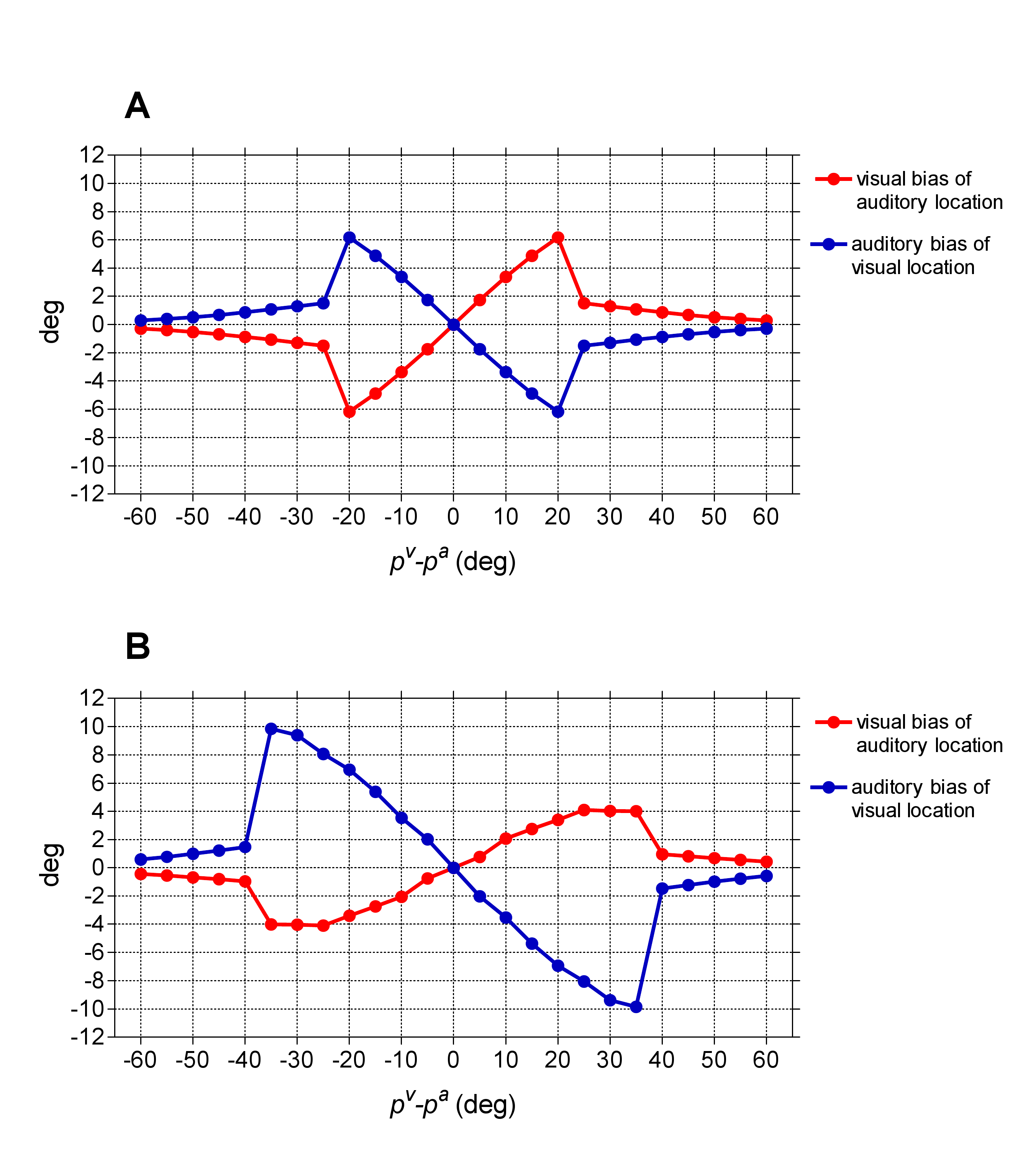

Supplement: Figure S2 — Effect of degrading visual spatial information. This figure is at integration of the sensitivity analysis, in particular it integrates Fig. 5H. (A) Visual bias of sound location and auditory bias of visual location were computed when the standard deviation of the external visual stimulus equals that of the auditory stimulus (σv = σa = 32°). All other network parameters are maintained at their basal value. When the two stimuli are sufficiently close (distance below 20–25°), the two stimuli affect reciprocally by the same extent. (B) Visual bias of sound location and auditory bias of visual location were computed when the standard deviation of the external visual stimulus was set greater than that of the auditory stimulus (σv = 40°; σa = 32°). All other network parameters are maintained at their basal value. Sound exerts a strong capture effect on the visual stimulus, which exhibits a shift as large as 10° towards the sound location; conversely, sound is only moderately attracted by the visual stimulus. (TIF) [file pone.0042503.s002.tif]

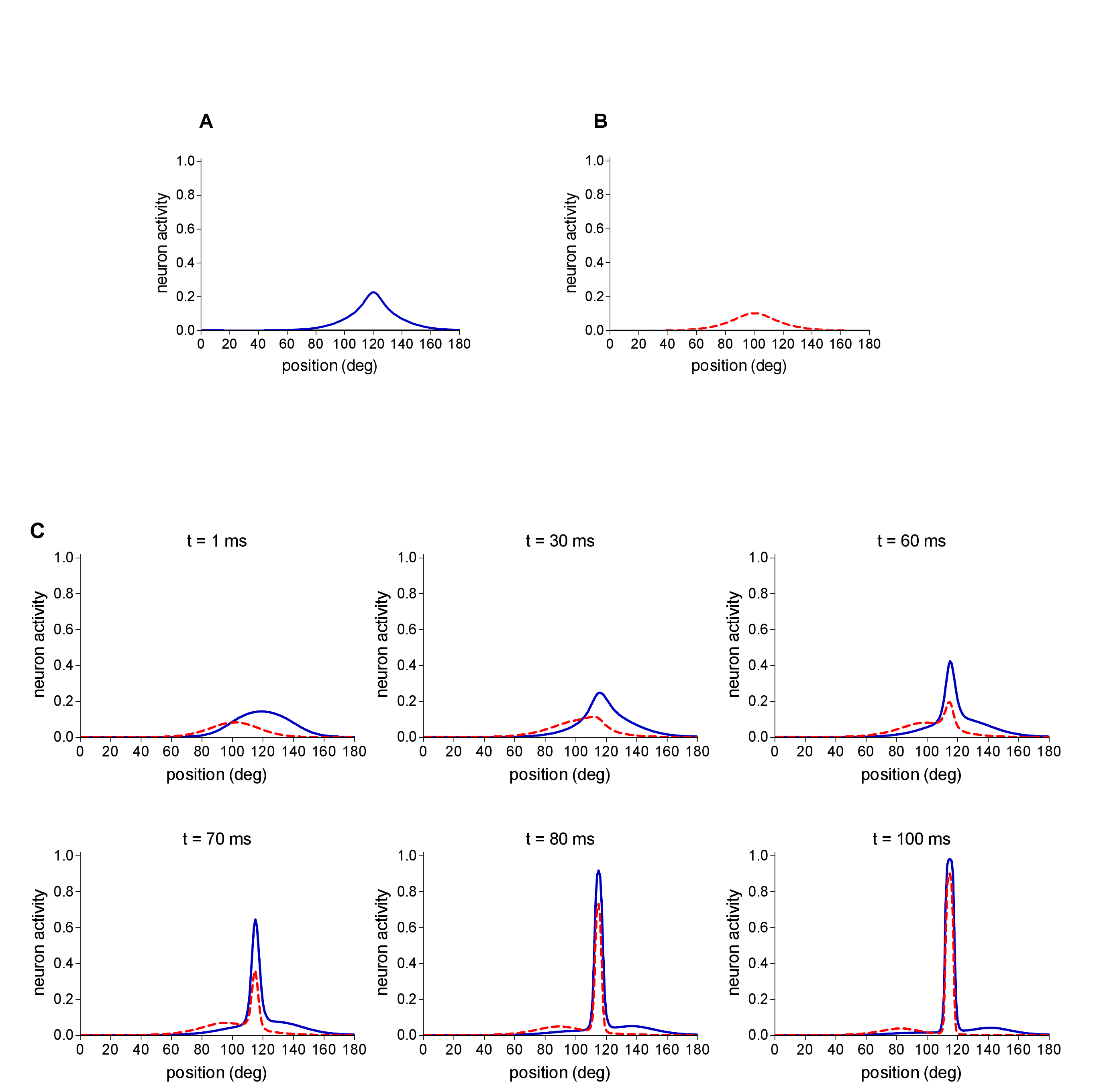

Supplement: Figure S3 — Alternative coding of cue reliability. Here we tested network functioning when cue reliability is coded in the strength (rather than the width) of the input. That is, here we assumed that the visual and auditory inputs have the same standard deviation (σv = σa = 35°) but different strengths. All other mechanisms and parameters have been maintained unaltered. The three parts of the figure display visual (blue line) and auditory (red dashed line) activity in response to different stimulations. (A) Network activity in response to an unimodal visual stimulus applied at position pv = 120°. The visual stimulus had strength = 16 (σv = 35°). The displayed activation refers to steady-state condition (after the transient response was exhausted). (B) Network activity in response to an unimodal auditory stimulus applied at position pa = 100°. The auditory stimulus had strength = 12 (σa = 35°). The displayed activation refers to steady-state condition (after the transient response was exhausted). (C) Different snapshots of network activity at different instants during the presentation of two cross-modal stimuli in spatial disparity (pv = 120°, pa = 100°). The visual and the auditory stimuli were the same as those presented in panels A and B (that is having same widths but different strengths). The auditory activity and the visual activity tend to reinforce reciprocally owing to the inter-area synapses; due to the higher strength of the visual input, visual activity around 120° is advantaged and shows a higher increase, amplifying auditory activation at this same position. At the new steady state (t≥100 ms), the perceived position of the auditory stimulus exhibits a strong shift towards the visual stimulus location (perceived position = 109.4°), whereas the shift of the visual stimulus is moderate (perceived position = 117.5°). (TIF) [file pone.0042503.s003.tif]
